# Women in Neurosurgery: Historical Path to Self-Segregation and Proposal for an Integrated Future

**DOI:** 10.3389/fsurg.2022.908540

**Published:** 2022-06-28

**Authors:** D. Garozzo, R. Rispoli, F. Graziano, R. M. Gerardi, A. Grotenhuis, A. Jenkins, V. Sammons, M. Visocchi, S. Pinazzo, R. Lima, F. Martinez, M. Emamhadi, M. T. Pedro, H. S. Shirwari, F. Guedes, I. D. Bhagavatula, D. P. Shukla, I. D. Bhat, O. A. Ojo, A. Tirsit, M. E. Gonzales-Gonzales, F. Luna, T. Kretschmer, E. Benzel, B. Cappelletto

**Affiliations:** ^1^ Department of Neurosurgery, Mediclinic Parkview Hospital, Dubai, UAE; ^2^ SOC Chirurgia Vertebro-Midollare, Azienda Sanitaria Universitaria Friuli Centrale, Presidio Ospedaliero Universitario Santa Maria della Misericordia di Udine, Udine, Italy; ^3^ Department of Neurosurgery, ARNAS Garibaldi Hospital, Catania, Italy; ^4^ Department of Neurosurgery, University Hospital, Palermo, Italy; ^5^ Department of Neurosurgery, Radboud UMC, Nijmegen, The Netherlands; ^6^ Department of Neurosurgery, Royal Victoria Infirmary, Newcastle upon Tyne, United Kingdom; ^7^ Department of Neurosurgery, North Shore Private Hospital, Sydney, Australia; ^8^ Department of Neurosurgery, Agostino Gemelli IRCCS, Rome, Italy; ^9^ Department of Neurosurgery, Hospital Maciel, Montevideo, Uruguay; ^10^ Department of Neurosurgery, Hospital de Clínicas, Montevideo, Uruguay; ^11^ Brachial plexus and peripheral nerve injury center, Guilan University of Medical Sciences, Rasht, Iran; ^12^ Peripheral Nerve Unit, Department of Neurosurgery, BKH Günzburg at Ulm University, Günzburg, Germany; ^13^ Department of Neurosurgery, Dawodzai Medical Complex, Jalalabad, Afghanistan; ^14^ Division of Neurosurgery, Gaffrée e Guinle University Hospital, Federal University of the State of Rio de Janeiro, Rio de Janeiro, Brazil; ^15^ Department of Neurosurgery, NIMHANS, Bangalore, India; ^16^ Department of Neurosurgery, RV Astor Hospital Sarakki Jp Nagar, Bengaluru, India; ^17^ Department of Surgery, College of Medicine, University of Lagos, Lagos, Nigeria; ^18^ Department of Neurosurgery, Addis Ababa University, Addis Ababa, Ethiopia; ^19^ Department of Neurosurgery, Hospital Civil de Guadalajara, Guadalajara, Jalisco, Mexico; ^20^ Departament of Neurosurgery, Hospital Clínico Regional de Concepción, Universidad de Concepción. Concepcion, Chile; ^21^ Dept. of Neurosurgery & Neurorestoration, Neurosurgical Intensive Care, Neurooncological Centre (DKG) Klinikum Klagenfurt, Klagenfurt, Austria; ^22^ Department of Neurosurgery, Cleveland Clinic, Cleveland, OH, United States of America

**Keywords:** women in neurosurgery, gender discrimination, gender equality, female under-representation, self-segregation, medical education

## Abstract

Despite the rising percentage of women accessing the medical profession over the last few decades, surgical specialties are still largely male-dominated; in particular, a remarkable gender disparity is evident in neurosurgery, where only 19% of practitioners are females. Although women may be reluctant to choose a challenging specialty like neurosurgery due to concerns around how to balance family and career, it must be admitted that prejudices against female neurosurgeons have been deeply rooted for long, prompting many to give up and switch track to less demanding subspecialties. Among those who have persisted, many, if not most, have experienced difficulties in career progression and received unequal treatment in comparison with their male counterparts. In 1989, a group of 8 female neurosurgeons founded Women in Neurosurgery (WINS), an organization that aimed to guarantee inclusivity in neurosurgery, encouraging a better and more egalitarian working environment. Thereafter, WINS sessions were regularly promoted at international conferences, offering female neurosurgeons a platform to report issues related to gender discrimination. Over recent years, the mission of WINS sessions in national and international conferences has taken an unexpected deviation; they have progressively become supplementary scientific sessions with only women neurosurgeons as speakers, thus paving the road to a form of self-segregation. This tendency has also resulted in the establishment of sections of only female neurosurgeons within some national societies. Although there remains a faction that fiercely supports the WINS mindset of reserved spaces for women, such segregation is an upsetting prospect for those who believe that science and professionalism have no gender; a growing part of the global neurosurgical community believes that the conception of a “female neurosurgery” and a “male neurosurgery” is misguided and counterproductive and consider the existence of the WINS as anachronistic and no longer necessary.

## Introduction

The World Health Organization collects global data on the proportion of women employed as physicians; despite variability in terms of the quality of data and the reference year, the organization provides a useful international comparison across Europe and for other countries with a total physician workforce >20,000. Based on most data collected during the early 2000s and in Europe, the mean proportion of women working as physicians was equal to 40% (SD 8.8) (1). Over the last two decades, the female presence in medical schools and residency programs has further increased; in the United States, for instance, in the last 5 years women have come to exceed men in the number of medical school applications and matriculation (2). In Australia and New Zealand this has been the case for over 10 years, with a greater proportion of medical students being female. (https://anzsurgsocs.org/wp-content/uploads/2021/01/gender_equity_guide.pdf)

Despite the rising percentage of women entering the medical profession, surgical specialties remain universally male-dominated. This gender disparity is particularly evident in trauma and orthopedic surgery, cardiothoracic surgery and in neurosurgery.

Neurosurgery is a modern surgical subspecialty; attempts to perform surgery for diseases affecting the nervous system already date back to the dawn of civilization (3–9) and have marked the historical development of medicine, however neurosurgery only became a well-defined surgical subspecialty at the beginning of the 1900, thanks to Harvey Cushing (10, 11).

Female entry to neurosurgery is not clearly historically dated. In the 1920′s, in Eastern Europe, neurosurgical procedures were allegedly performed by female surgeons, namely Anna Bormane, Serafima Semyonovna Bryusova and Alice Rosenstein (11, 12). In 1943, in the United Kingdom, Diana Beck was appointed consultant neurosurgeon at the Royal Free Hospital in London, thus becoming the first formally certified female neurosurgeon; although this record is often attributed to the Romanian Sofia Ionescu, she did not qualify from medical school until 1945 (13, 14). Thereafter, several women received neurosurgical training around the world, although female access to this subspecialty remained occasional, especially in countries characterized by traditionally male-dominated societies. Until recent times, women have occupied fewer than 10% of residency positions, thus being too few to be even considered a “minority” (defined as 15%) or create a so-called critical mass, large enough to independently attract other females. Trends from the last 10 years indicate that the number of female neurosurgeons is growing steadily, however women remain a minority in the field and currently account for only 19% of all board-qualified neurosurgeons globally (15).

Several factors may be posited to explain this remarkable gender disparity in neurosurgery (16). For instance, many women may have been reluctant to choose neurosurgery, being concerned about how to balance family and career. In a survey study of 245 fourth-year students at the University of Toronto, sampling qualities of importance in specialties, the importance of role models and attitudes toward surgery, males were more likely to identify technical challenge, earning potential, and prestige (*P* < 0.01) as important qualities in a specialty whereas females tended to focus on residency conditions, part-time work, and parental leave availability (*P* < 0.01) (17). Especially in countries where the burden of children's upbringing is traditionally placed on females, many women spontaneously give up or are encouraged to switch track to less demanding subspecialties (17–23). In a survey on the status of women neurosurgeons in India, 72.68% of the participants reported that they had been discouraged before joining neurosurgical residency (24). Women also frequently have to choose between family and career; female neurosurgeons are less likely to be married and to have children than their male counterparts (22–27). There is also evidence that they experience higher rate of failed births during residency (26).

While it is true that women may be reluctant to choose a challenging specialty like neurosurgery due to concerns about balancing family and career, prejudice against female neurosurgeons—whether conscious or unconscious—remains deeply rooted. A subtle selection bias is often present when it comes to mentoring a female candidate in the residency program, especially more apparent in the developing countries where the attitude of males towards females is judgmental and not inclusive (28). Besides the fear that embracing motherhood might impact their work performance, female neurosurgeons have often been considered less “suitable” for the profession, as they have been attributed alleged traits of “weakness” and “lack of physical resistance”, as openly declared in 2005 by a very well-known neurosurgeon, who had also been president of SINCH (the Italian Society of Neurosurgery), in an interview in “Corriere della Sera” (the Italian newspaper with the highest circulation in the country) (29).

Many of those who persist experience difficulties in career progression and receive unequal treatment in comparison with their male counterparts (26, 27, 30, 31), garnering less trust and respect from patients and colleagues (22, 26, 27); there is also a lack of acceptance of female leadership (22, 27, 32, 33).

In various surgical subspecialties, women are more often assigned administrative tasks while men might have more opportunities to operate (34). Some women surgeons had difficulty in finding consultant jobs because there is prejudice amongst seniors against women leading or being consultants (35). Many women have reported that they have to work harder to prove that they were as good as men and have received less career support from supervisors and acceptance in neurosurgical communities. This is mirrored in the gross under-representation of women in leadership roles. Italy provides an excellent example; although it currently boasts the highest proportion of female neurosurgeons in Europe (36% of Italian neurosurgeons), the representation of females amongst the higher ranks is ridiculously negligible (32, 36). Only 2 out of 138 neurosurgery department in the country are led by a woman, only one full-time professor is female (the overall number of neurosurgery professor is around 60) and only one woman is currently member of the board of the national neurosurgical society. Similar under representation is found also in other societies (37, 38) for instance, only 12% of the Deutsche Gesellschaft für Neurochirurgie—German Society of Neurosurgery (DGNC) members are female and no member of the current board for the term 2020–2022 is a woman (39).

Another example is in Uruguay where 12 out of 37 neurosurgeons are females, recording the highest percentage of female neurosurgeons in South America (36) but no female neurosurgeon is full-time professor; three neurosurgical departments are led by two women, one of them is in a military hospital where the appointment was not subjected to open tender but achieved as progression in the military career and the remaining two (both under the same leadership) are private institutions.

## The Academic Scenario

Literature exploring the productivity of women in neurosurgery found no difference in h-index when matched with male neurosurgeons of the same academic rank, as shown by Khan et al. in their study on publication productivity measures for almost all academic neurosurgeons and departments in the United States (40). In a study published by Aslan et al., the data of 3,247 issues of Neurosurgery and Journal of Neurosurgery published in 2003, 2008, 2013, and 2018 were analyzed, determining the gender of the first and last authors in all articles. The overall percentage of female first authors was 16% (518/3,247), whereas that of female senior authors was 10.8% (352/3,247). The percentage of women first authors increased from 12% to 16.5% in 5 years (from 2003 to 2018), although regarding the last authorship, the rates of women rates remained unchanged over the years (41). Despite their increasing publication productivity, the representation of women on editorial boards of neurosurgery is very low and appears to be even lower in spine surgery (42).

Women continue to encounter difficulties in accessing the academic world (32, 33, 43). Even when adjusted for hours worked, they are under referred patients and are also less likely to be promoted and achieve a higher academic ranking (44). Databases from the American Association of Neurological Surgeons (AANS) and the American Board of Neurological Surgery (ABNS) from 1964 to 2013 were reviewed for female neurosurgery residency graduates. Of 379 female neurosurgery resident graduates, about 26% entered academic medicine, which is similar to analogous data for the male counterparts; however, only 33 women (8%) managed to attain the rank of associate professor, and only 16 (4%) reached the rank of professor, a staggering drop-off compared with the positions achieved by male neurosurgeons (43).

Scientific events are also often characterized by an uneven distribution between female and male speakers and female chairs are occasional. With a few exceptions, chances to present work or chair a session have been considerably reduced for women compared to men. Furthermore, female speakers are less likely to give presentations typically associated with senior positions such as plenary sessions or keynote presentations (45); it has been noted that, in the annual conferences of the above mentioned DGNC, the speaking time of women was distinctly shorter than that of men, and women were more often assigned to the less popular poster presentations.

## The Fight for Equality: Birth and Historical Development of Wins

In 1989, during a meeting in Atlanta a group of 8 female neurosurgeons founded “Women in Neurosurgery” (WINS), an organization with the mission to “educate, inspire, and encourage women neurosurgeons to realize their professional and personal goals, and to serve neurosurgery in addressing the issues inherent to training and maintaining a diverse and balanced workforce.” Deborah Benzil was the first President of the organization. In 2008, they penned a white paper describing four strategies to address the lack of women in neurosurgery (46):

(1) characterize barriers;
(2) identify and eliminate discriminatory practices when recruiting medicals students, training residents, and hiring and promoting of neurosurgeons;
(3) promote women into leadership positions within organized neurosurgery;
(4) foster the development of female neurosurgeon role models by training and promoting competent female trainees and surgeons.

In the years following its foundation, the initial cell of WINS gradually developed into a worldwide network that gathered under its umbrella female neurosurgeons who felt their gender had negatively impacted on their professional growth and who were eager to break down the barriers of male chauvinism, in order to create a neurosurgical community that would offer equal opportunities to all its members.

Female neurosurgeons who had succeeded in gaining international recognition of their merits became torchbearers in the march towards gender equality in neurosurgery. Japan's first female neurosurgeon Yoko Kato passionately dedicated herself to the cause of female empowerment in neurosurgery and founded the Women's Neurosurgical Association of Japan in 1990 and the Asian Women's Neurosurgical Association in 1996 (47).

In 2008, the Korean Women Neurosurgical Society was founded, under the lead of Hyo-Sook Chung, first woman neurosurgeon in South Korea (48).

In 2014, the WINS section of the Brazilian Society of Neurosurgery was founded under the coordination of Nelci Zanon, soon afterwards followed by the Colombian WINS. The Venezuelan WINS was founded years later, in early 2020 (49). In 2015, Aneela Darbar founded WINS Pakistan; WINS India forum was then inaugurated in 2016 under the presence of Thanjavur Santhanakrishna (T.S.) Kanaka, who was the first woman neurosurgeon in Asia in1968. Taiwan WINS was established in 2017 while in China, Ling Feng established WINS of China uniting over 200 female neurosurgeons (50).

WINS sections were established in many national and international, umbrella societies, such as FLANC (the Latin American Federation of Neurosurgery), CAANS (Continental Association of African Neurosurgical Societies) and even in WFNS (World Federation of Neurosurgical Societies)

The idea of WINS was brilliant at birth. First, it brought a reality to light: the existence of women neurosurgeons. It might currently sound outdated due to the remarkable increase in the number of female neurosurgeons in the world, yet one should not forget that female emancipation is an achievement that only dates back to a few decades ago; it is still a difficult process in many parts of the world where society is traditionally male-dominated, and women's freedom is greatly limited by the boundaries that these communities impose on them. If the “glass ceiling” is an issue still perceived even in the most gender-egalitarian countries (37), there are countries in the world where even the access of women to education is questioned (https://www.researchgate.net/publication/323497891_Female_Education_in_Developing_countries); choosing to embrace a demanding career as neurosurgery is truly a social challenge in such sociocultural contexts.

WINS provided a voice to hundreds of professionals and offered them a useful platform in which to report issues related to gender discrimination (28). However, over the years WINS sessions have progressively become supplementary scientific sessions with only female neurosurgeons as speakers. In the Covid-19 era, where virtual scientific events were the only solution to replace in person gatherings, female speakers- only webinars flourished.

This deviation stemmed from praiseworthy intentions, to highlight, promote and consolidate the clinical and scientific activity of women in neurosurgery (28), whose role in conferences is frequently marginal, as previously mentioned. Moreover, WINS specifically aimed to encourage women towards clinical research, especially in low- and middle-income countries (28), where the gender bias is heavier (51).

There is a contrary view, however, that reserving separate spaces for female speakers in neurosurgical conferences may be dangerous; it can easily pave the road to self-segregation.

Despite the laudable intentions of its supporters, this kind of organization further raises the barriers between the two genders; female speakers are actually relegated to pink rooms and, instead of being integral to the neurosurgical community, become even more marginalized. Men who have previously fostered integration may be pushed into the “male section” of the profession and implicit labels of male chauvinism may unfairly be attached to those men who had naturally interacted with their female colleagues in a peer-to-peer relationship. Although there remains a non-negligible faction that fiercely supports the WINS mindset of reserved spaces for women, such segregation is an upsetting prospect for those who believe that science and professionalism have no gender; a growing part of the global neurosurgical community believes that the conception of a “female neurosurgery” and a “male neurosurgery” is misguided and counterproductive.

During his term as EANS president, Grotenhuis, raised concerns about the development of a section of Women in Neurosurgery within the European Association of Neurosurgical Societies (EANS) as advocated by 66% of female neurosurgeons in a survey study in 2019 (3723). Wolfert et al*.* analyzed female participation in EANS and its member societies and also conducted an online questionnaire on career choice, mentorship, family planning, and gender discrimination. The 116 responses that were received highlighted that most female neurosurgeons complained of the lack of same-gender role models (76%), emphasized the importance of having a female mentor (58%) and considered that their greatest obstacle was the prevailing inequality in opportunities (30%) and attaining leadership positions (24%). In his letter to the WFNS, although Grotenhuis confirmed being sadly aware of the discrimination that female neurosurgeons would still experience both in their practice and academic career (as shown in the study by Wolfert et al.), he believed that the foundation of a WINs section in the EANS would overshadow the simple and obvious fact that we are all neurosurgeons and it would only end up highlighting one's gender more than their professionalism, therefore separating rather than integrating female neurosurgeons in the global neurosurgery community. Instead of a WINS section a task-force on diversity in a broader sense was established in the EANS (52) and two of the authors of the initial study were later appointed to leadership roles within it (27).

## The Role of Wins Today

Female emancipation commenced less than a century ago and is still ongoing; in many parts of the world, a woman is still not granted the same rights as a man.

Historically, women's subordination has been justified by attributing to them traits of weakness, lack of physical endurance, and even inferior cognitive skills. Despite the evident and undeniable biological differences between the two genders, it is now clear that these prejudices have no justification.

Male chauvinism is still pervasive in society and has tainted the neurosurgical community as well, drawing on the same misconceptions and prejudices that have burdened women through history. Nevertheless, it is undeniable that the last few decades have been characterized by considerable political and socio-economic upheaval; things have changed a lot, including neurosurgery. There is a tangible and growing ambition in women in the neurosurgical field, from medical student to attending level, and a significant number of women has succeeded in accessing leadership roles in national and international neurosurgical societies. For instance, two of the most relevant position in the EANS (Chair of the Individual Membership Committee and Chair of the Young Neurosurgeons Committee) are currently held by women (https://www.eans.org/page/Committees). Even traditionally male-dominated social communities are touched by the wind of change; in Rwanda, the first and only female neurosurgeon is running a neurosurgery unit at the Rwanda Military Hospital (RMH), one on the country's largest tertiary referral hospitals (53).

Perhaps the most significant demonstration of this ongoing change has been the election of Najia El Abbadi as president elect of the World Federation of Neurosurgical Societies (WFNS) for the period 2021–2023. She will be the first woman at the head of the organization that incorporates all national societies and has been elected by both male and female neurosurgeons around the world. This election is an indicator of the revolutionary changes in the collective neurosurgical mindset that can lead to the abolition of a cumbersome organization that nowadays merely contributes to gender discrimination instead of fostering integration.

Finally, the Covid-19 pandemic has further contributed to the promotion of women's participation in neurosurgical meetings. Due to the introduction of virtual workshops (webinars) to replace in-person international meetings in order to highlight neurosurgical activities worldwide during such a critical time, the role and merit of female professionals became evident both in research and surgical practice; in about 30 webinars organized by WFNS Neuro-rehabilitation and Reconstructive Committee, the number of women as invited speakers recently increased up to 50% in comparison with male speakers (54).

We will only be able to make the neurosurgical community immune from gender discrimination when it is completely erased from society. Education is the only way to build an inclusive society where discrimination has no place; in order to accomplish this seismic shift, the new values must be seeded in the minds of the young generations from their early childhood.

In the meantime, if discrimination persists in neurosurgery, the burden of fighting against it must be equally shared by both male and female neurosurgeons. In order to help women to successfully combine family and professional life, redesigning neurosurgical training and offering more flexibility in the curriculum might be a valid solution (26, 27, 28). The boards of each society (in particular WFNS) should supervise and enforce measures that establish equity. In harmonious collaboration with their male counterparts, female presence in the high ranks of the neurosurgical societies must ensure that no form of discrimination happens. Where an uneven distribution of presentations in a conference may be due to gender discrimination, the solution cannot be to reserve spaces for female speakers in pink rooms. Based on their professional value, the contribution of female neurosurgeons should be fully integrated in the scientific program of each conference; professionalism and merit are the only features that should attract attention. Young neurosurgeons of both genders living in low- and middle-income countries should be equally supported through scholarships in order to have access to scientific events The importance of mentorship has been highlighted (26, 27, 55–57); in this regard, women in leadership positions, aware of the difficulties that young females in their early career may experience, might be particularly committed to offer guidance to young professionals and help them to fully express their potential.

## Conclusion: Towards the Future

Gender discrepancy is a hot topic in the neurosurgical community; WINS contributed to create and promote the culture of change, playing a pivotal role in the fight against prejudice. Great strides were made in the past decade and further, major changes will take place in the near future. Currently however, the existence of WINS is anachronistic and should no longer be necessary in a new era, open to integration, inclusivity, and equality.

The continued development of neurosurgery can be achieved only through promotion of professionalism, regardless of the gender of those who practice this challenging surgical specialty.

Both men and women neurosurgeons actually encounter difficulties in their career. The focus of this paper is on gender yet other disparities (e.g., ethnic) burden the professional progression of many neurosurgeons and should also be recognized. Self-segregation is not the solution. On the contrary, we need to work together to change workplace culture, creating an environment that enables all neurosurgeons to have a successful professional and personal life and where diversity becomes an element of strength and not weakness.

## Data Availability

The original contributions presented in the study are included in the article/Supplementary Material, further inquiries can be directed to the corresponding author/s.

## References

[1] World Health Organisation. Global Atlas of the Health Workforce: Gender Distribution of Selected Health Professions. Geneva (2006).

[2] Yaeger KA Munich SA Byrne RW Germano IM. Trends in United States neurosurgery residency education and training over the last decade (2009–2019). Neurosurgery. (2020) 48:E6. 10.3171/2019.12.FOCUS19827 32114562

[3] González-Darder JM. La trepanación craneal en las culturas primitivas [Cranial trepanation in primitive cultures]. Neurocirugia (Astur). (2017) 28(1):28–40. 10.1016/j.neucir.2016.04.003 27208912

[4] Collado-Vázquez S Carrillo JM. Cranial trepanation in the Egyptian. Neurología (English Edition). (2014) 29(7):433–40. 10.1016/j.nrleng.2011.05.008 21889823

[5] Carod-Artal FJ Vázquez-Cabrera CB. Paleopatología neurológica en las culturas precolombinas de la costa y el altiplano andino (II). Historia de las trepanaciones craneales [Neurological paleopathology in the pre-Columbine cultures of the coast and the Andean plateau (II). The history of cranial trepanations]. Rev Neurol. (2004) 38(9):886–94. 10.33588/rn.3809.2004066 15152360

[6] Andrushko VA Verano JW. Prehistoric trepanation in the Cuzco region of Peru: a view into an ancient Andean practice. Am J Phys Anthropol. (2008) 137(1):4–13. 10.1002/ajpa.20836 18386793

[7] Lv X Li Z Li Y. Prehistoric skull trepanation in China. World Neurosurg. (2013) 80(6):897–9. 10.1016/j.wneu.2012.08.009 23022649

[8] Papagrigorakis MJ Toulas P Tsilivakos MG Kousoulis AA Skorda D Orfanidis G Neurosurgery during the Bronze Age: a skull trepanation in 1900 BC Greece. World Neurosurg. (2014) 81(2):431–5. 10.1016/j.wneu.2013.01.044 23321381

[9] Arena F Larocca F Gualdi-Russo E. Cranial surgery in Italy during the bronze age. World Neurosurg. (2022) 157:36–44. 10.1016/j.wneu.2021.09.105 34607065

[10] Savitz SI. The pivotal role of Harvey Cushing in the birth of modern neurosurgery. JAMA. (1997) 278(13):1119. 10.1001/jama.278.13.1119 9315778

[11] Spetzler RF. Progress of women in neurosurgery. Asian J Neurosurg. (2011) 6(1):6–12. 10.4103/1793-5482.85627 22059098 PMC3205553

[12] Hernández-Durán S Kim E Ivan D Rosseau G Murphy M. Four Athenas- Europe’s first female neurosurgeons. J Clin Neurosci. (2021) 86:332–36. 10.1016/j.jocn.2021.01.033 33558183

[13] Gilkes CE. An account of the life and achievements of Miss Diana Beckneurosurgeon (1902-1956). Neurosurgery. (2008) 62(3):738–42. 10.1227/01.neu.0000317324.71483.e5 18425021

[14] Duncan N. Caring physicians of the world. New York: Pfizer Medical Human Initiatives (2005). p. 67.

[15] Steklacova A Bradac O De Lacy P Benes V. E-WIN project 2016: evaluating the current gender situation in neurosurgery across Europe-an interactive, multiple-level survey. World Neurosurg. (2017) 104:48–60. 10.1016/j.wneu.2017.04.094 28456744

[16] Graziano F Gerardi RM Scalia G Cammarata G Nicoletti GF Chaurasia B Women in neurosurgery: from a matter of fortuitous occasions toward a conscious choice. World Neurosurg. (2021) 148:129–35. 10.1016/j.wneu.2021.01.049 33515798

[17] Baxter N Cohen R McLeod R. The impact of gender on the choice of surgery as a career. Am J Surg. (1996) 172:373–76. 10.1016/S0002-9610(96)00185-7 8873533

[18] Marks IH Diaz A Keem M Ladi-Seyedian SS Philipo GS Munir H Barriers to women entering surgical careers: a global study into medical student perceptions. World J Surg. (2020) 44(1):37–44. 10.1007/s00268-019-05199-1 31616970

[19] Karekezi C Thango N Aliu-Ibrahim SA Bechri H You Broalet EM Bougrine M History of African women in neurosurgery. Neurosurg Focus. (2021) 50(3):E15. 10.3171/2020.12.FOCUS20905 33789234

[20] Ng CWQ Syn NL Hussein RBM Ng M Kow AWC. Factors attracting or deterring female medical students in Asia from pursuing a surgical career, and the impact of surgical clerkship, mentorship, and role models: a multicultural Asian perspective from a national prospective cohort study. J Surg Res. (2021) 260:200–9. 10.1016/j.jss.2020.11.053 33360303

[21] Klifto KM Payne RM Siotos C Lifchez SD Cooney DS Broderick KP Women continue to be underrepresented in surgery: a study of AMA and ACGME data from 2000 to 2016. J Surg Educ. (2020) 77:362–8. 10.1016/j.jsurg.2019.10.001 31889693

[22] Gadjradj PS Matawlie RHS Voigt I Harhangi BS Vleggeert-Lankamp CLAM. Gender differences between male and female neurosurgeons: is there equality for all? World Neurosurg. (2020) 136:348–56. 10.1016/j.wneu.2019.11.178 31821909

[23] Maehara T Kamiya K Fujimaki T Matsumura A Hongo K Kuroda S A questionnaire to assess the challenges faced by women who quit working as full-time neurosurgeons. World Neurosurg. (2020) 133:331–42. 10.1016/j.wneu.2019.08.045 31437517

[24] Palanisamy D Battacharjee S. What it is to be a woman neurosurgeon in India: a survey. Asian J Neurosurg. (2019) 14(3):808–14. 10.4103/ajns.AJNS_142_19 31497106 PMC6703048

[25] Fujimaki T Shibui S Kato Y Matsumura A Yamasaki M Date I Gender equality committee of the Japan neurosurgical society. Working conditions and lifestyle of female surgeons affiliated to the Japan neurosurgical society: findings of individual and institutional surveys. Neurol Med Chir (Tokyo). (2016) 56(11):704–8. 10.2176/nmc.oa.2016-0119 27302300 PMC5221781

[26] Thum JA Chang D Tata N Liau LM. Neurosurgeons in 2020: the impact of gender on neurosurgical training, family planning, and workplace culture. Neurosurg Focus. (2021) 50(3):E11. 10.3171/2020.12.FOCUS20965 33789233

[27] Shi HH Westrup AM O’Neal CM Hendrix MC Dunn IF Gernsback JE. Women in neurosurgery around the world: a systematic review and discussion of barriers, training, professional development, and solutions. World Neurosurg. (2021) 154:206–13. 10.1016/j.wneu.2021.07.037 34280544

[28] Balasubramanian SC Palanisamy D Bakhti S El Abbadi N Collange NZ Karekezi C Women in neurosurgery (WIN): barriers to progress, world WIN directory and the way forward. Asian J Neurosurg. (2020) 15(4):828–32. 10.4103/ajns.AJNS_108_20 33708650 PMC7869302

[29] Broggi G. Giovani medici, le donne raddoppiano gli uomini. Interview on Corriere della Sera: 22 (2005).

[30] Sandoval-Bonilla BA La Cerda-Vargas MF Stienen MN Nettel-Rueda B Ramírez-Reyes AG Soriano-Sánchez JA Discrimination of residents during neurosurgical training in Mexico: results of a survey prior to SARS-CoV-2. Surg Neurol Int. (2021) 12:618. 10.25259/SNI_813_2021 34992934 PMC8720478

[31] Seemann NM Webster F Holden HA Moulton CA Baxter N Desjardins C Women in academic surgery: why is the playing field still not level? Am J Surg. (2016) 211(2):343–9. 10.1016/j.amjsurg.2015.08.036 26723836

[32] Zhuge Y Kaufman J Simeone DM Chen H Velazquez OC. Is there still a glass ceiling for women in academic surgery? Ann Surg. (2011) 253(4):637–43. 10.1097/SLA.0b013e3182111120 21475000

[33] Lauretti L. Letter: tracking career paths of women in neurosurgery. Neurosurgery. (2019) 84:E92–3. 10.1093/neuros/nyy477 30365019

[34] Barnes KL McGuire L Dunivan G Sussman AL McKee R. Gender bias experiences of female surgical trainees. J Surg Educ. (2019) 76(6):e1–e14. 10.1016/j.jsurg.2019.07.024 31601487

[35] De La Peña NM Richter KR Haglin JM Pollock JR Richter RA Kouloumberis PE. Differences by practice year in numbers of U.S. female neurosurgeons. World Neurosurg. (2021) 145:363–67. 10.1016/j.wneu.2020.10.045 33068801

[36] Lulla T Behmer Hansen RT Smith CA Silva NA Patel NV Nanda A. Women neurosurgeons around the world: a systematic review. Neurosurg Focus. (2021) 50(3):E12. 10.3171/2020.12.FOCUS20902 33789239

[37] Wolfert C Rohde V Mielke D Hernández-Durán S. Female neurosurgeons in Europe-on a prevailing glass ceiling. World Neurosurg. (2019) 129:460–66. 10.1016/j.wneu.2019.05.137 31132491

[38] Forster MT Behrens M Lawson McLean AC Nistor-Gallo DI Weiss M Maurer S. Gender disparity in German neurosurgery. J Neurosurg. (2021) 136(4):1141–46. 10.3171/2021.3.JNS21225 34507274

[39] McLean AL. Women in German neurosurgery: status and representation at annual national meetings. Acta Neurochir. (2020) 162:231–36. 10.1007/s00701-019-04164-0 31848790

[40] Khan NR Thompson CJ Taylor DR Venable GT Wham RM Michael LM 2nd An analysis of publication productivity for 1225 academic neurosurgeons and 99 departments in the United States. J Neurosurg. (2014) 120(3):746–55. 10.3171/2013.11.JNS131708 24359012

[41] Aslan A Kuzucu P Karaaslan B Börcek AÖ. Women in neurosurgery: gender differences in authorship in high-impact neurosurgery journals through the last two decades. World Neurosurg. (2020) 138:374–80. 10.1016/j.wneu.2020.03.017 32200013

[42] Ramos MB Criscuoli de Farias FA Einsfeld Britz JP De Quadros FW Koch KB Carobin VN Representation of women on editorial boards of medline-indexed spine, neurosurgery, and orthopedic journals. Int J Spine Surg. (2022) 16(2):404–11. 10.14444/8223 35444047 PMC9930665

[43] Renfrow JJ. Tracking career paths of women in neurosurgery. Neurosurgery. (2018) 82(4):576–82. 10.1093/neuros/nyx251 28521026

[44] Finn R Prisco L Ganau M. Improving gender equality in the surgical workplace. JAMA Surg. (2020) 155(5):448–49. 10.1001/jamasurg.2019.6026 32049267

[45] Benzil DL Von Der Schmidt E 3rd. Toward harnessing forces of change: assessing the neurosurgical workforce. AANS Bulletin. (2006) 15:7–11.

[46] Benzil DL Abosch A Germano I Gilmer H Maraire JN Muraszko K The future of neurosurgery: a white paper on the recruitment and retention of women in neurosurgery. J Neurosurg. (2008) 109(3):378–86. 10.3171/JNS/2008/109/9/0378 18759565

[47] Kutty RK Mundhe V. Burden of dengue related neurosurgical emergency. Asian J Neurosurg. (2019) 14(1):211–18. 10.4103/ajns.AJNS_318_17 30937037 PMC6417330

[48] Jung TY Kim EY Park MS. History of the Korean women neurosurgical society since 2008. J Korean Neurosurg Soc. (2019) 62:619–25. 10.3340/jkns.2019.0043 31295978 PMC6835153

[49] Zanon N Niquen-Jimenez M Kim EE Zegarra AB Ramírez-Reyes AG Quiroga DP Progress in neurosurgery: contributions of women neurosurgeons in Latin America. J Clin Neurosci. (2021) 86:347–56. 10.1016/j.jocn.2021.01.054 33653668

[50] Drummond KJ Kim EE Apuahe E Darbar A Kedia S Kuo MF Progress in neurosurgery: contributions of women neurosurgeons in Asia and Australasia. J Clin Neurosci. (2021) 86:357–36. 10.1016/j.jocn.2021.02.002 33618964

[51] Abosch A Rutka JT. Women in neurosurgery: inequality redux. J Neurosurg. (2018) 129(2):277–81. 10.3171/2018.4.JNS172878 29999441

[52] Grotenhuis A. Women in neurosurgery: A personal contemplation. Letter to WFNS (2020).

[53] Chu K. Inspirational women in surgery: dr claire karekezi, neurosurgeon, Rwanda. World J Surg. (2022). 10.1007/s00268-022-06490-4. Epub ahead of print. PMID: 35246713

[54] Mathiesen T Arraez M Asser T Balak N Barazi S Bernucci C A snapshot of European neurosurgery December 2019 vs. March 2020: just before and during the COVID-19 pandemic. Acta Neurochir. (2020) 162(9):2221–33. 10.1007/s00701-020-04482-8 32642834 PMC7343382

[55] Dixon A Silva NA Sotayo A Mazzola CA. Female medical student retention in neurosurgery: a multifaceted approach. World Neurosurg. (2019) 122:245–51. 10.1016/j.wneu.2018.10.166 30391758

[56] Renfrow JJ Rodriguez A Liu A Pilitsis JG Samadani U Ganju A Positive trends in neurosurgery enrollment and attrition: analysis of the 2000– 2009 female neurosurgery resident cohort. J Neurosurg. (2016) 124(3):834–39. 10.3171/2015.3.JNS142313 26452119

[57] Taha B Sadda P Winston G Odigie E Londono C Greenfield JP Increases in female academic productivity and female mentorship highlight sustained progress in previously identified neurosurgical gender disparities. Neurosurg Focus. (2021) 50(3):E3. 10.3171/2020.12.FOCUS20939 33789232

